# Cyclosporine in the Treatment of Drug Reaction With Eosinophilia and Systemic Symptoms Syndrome: Retrospective Cohort Study

**DOI:** 10.2196/41391

**Published:** 2023-07-20

**Authors:** Sophia Zita, Lindsey Broussard, Jeremy Hugh, Sabrina Newman

**Affiliations:** 1 Department of Dermatology University of Colorado Aurora, CO United States; 2 Department of Dermatology Stony Brook University Stony Brook, NY United States; 3 SkinMed Institute Lone Tree, CO United States

**Keywords:** drug reaction with eosinophilia and systemic symptoms, drug-induced hypersensitivity syndrome, drug reactions, eosinophils, cyclosporine, treatment, skin, rash, dermatology, drug reaction, adverse reaction, eosinophil, eosinophilia, Systemic Symptoms, drug-induced, drugs, cohort study, case series, pharmaceutic, pharmacology, pharmacy

## Abstract

**Background:**

Drug reaction with eosinophilia and systemic symptoms (DRESS) syndrome is a severe, life-threatening reaction to a culprit drug that frequently involves end-organ damage. Corticosteroids are the first-line treatment for DRESS syndrome; however, corticosteroids may be contraindicated in certain patient populations. There are currently only 54 cases detailing the use of cyclosporine for the treatment of DRESS syndrome reported in the literature.

**Objective:**

The aim of this case series was to examine the treatment of DRESS syndrome with cyclosporine in a large patient cohort by aggregating time to symptom resolution, recurrence rate, and treatment dose and duration.

**Methods:**

This study was a retrospective cohort study. Patients diagnosed with DRESS syndrome by a board-certified dermatologist and treated at the University of Colorado Hospital from 2015 to 2019 were included.

**Results:**

Our inclusion criterion was met by 19 occurrences of DRESS syndrome. With a short course of cyclosporine, 17 of 19 patients in our cohort (89%) had resolution of symptoms (mean treatment length of 5.26 days). DRESS syndrome’s relapse after treatment with cyclosporine occurred in 3 of 19 (16%) occurrences of the cohort.

**Conclusions:**

Our study supports the use of cyclosporine in the treatment of DRESS syndrome, particularly in patients who are unable to sustain prolonged immunosuppression. Further research is necessary to compare the efficacy of cyclosporine to the current standard of care in a larger study population and investigate long-term outcomes.

## Introduction

Drug reaction with eosinophilia and systemic symptoms (DRESS) syndrome causes severe cutaneous and systemic complications with a mortality rate of ≈10% [1,2]. The current standard of care for DRESS syndrome involves the removal of the culprit drug, supportive therapy, and systemic corticosteroids. Corticosteroid therapy must be tapered for weeks to months to prevent DRESS syndrome relapse [1]. Additionally, corticosteroid therapy may be contraindicated, induce adverse events (such as hyperglycemia and hypertension), and increase the risk of viral reactivation. To the best of our knowledge, there are only 54 previously reported cases of cyclosporine use in the treatment of DRESS syndrome [2-16]. Half of these previously reported cases (27 of 54) are reported in a retrospective study comparing cyclosporine to corticosteroid use in the treatment of DRESS syndrome, which found that there was no significant difference in time to resolution of DRESS syndrome between the 2 groups. However, there were more adverse events in the corticosteroid group compared to the cyclosporine group [12]. Of the remaining, 27 cases, 23 cases have demonstrated cyclosporine to be an effective alternative therapy to corticosteroids [2-11]. Despite these promising results, the number of cases reported in the literature remains low.

## Methods

### Participants

This retrospective review of medical records analyzed patients with DRESS syndrome who were treated with cyclosporine at the University of Colorado Hospital (UCH) from January 2015 to August 2019. Medical record numbers for patients with a diagnosis of DRESS syndrome who had received cyclosporine were collected using Health Data Compass, yielding 26 patients with 36 cases of DRESS syndrome. Researchers subsequently examined these patient charts to determine if each case of DRESS syndrome met the following inclusion criteria: a Registry of Severe Cutaneous Adverse Reaction (RegiSCAR) score >2 [17], age greater than 18 years, diagnosis of DRESS syndrome made by a board-certified dermatologist, and treatment with systemic cyclosporine for at least 3 days after DRESS syndrome diagnosis. The RegiSCAR score was calculated retrospectively using data from the electronic medical record, including laboratory results, imaging, physical exam findings, and clinical symptoms. This inclusion criterion was met by 17 patients with a total of 19 occurrences of DRESS syndrome. Of these 19 occurrences, 18 were treated in the inpatient setting. Recurrence of DRESS syndrome and follow-up time were determined by reviewing the University of Colorado Health System’s electronic medical record from initial DRESS syndrome occurrence through March 2022.

### Outcomes

The primary outcome of the study was to determine time to clinical resolution, which was defined as sustained temperature <38.5 °C with rash improvement, when treated with cyclosporine. Secondary outcomes included recurrence of DRESS syndrome, cyclosporine dose, and treatment length.

### Ethical Considerations

This study was reviewed and exempted by the Colorado Multiple Institutional Review Board.

## Results

### Participants

The mean RegiSCAR score of the 19 DRESS syndrome cases was 3.42. The patients were mostly male (11/19, 58%) with cases of DRESS syndrome occurring at an average age of 48.1 years. The most common causative agents were phenytoin and vemurafenib. Approximately 11 of 19 (58%) of the patients were treated with prednisone prior to admission at UCH or for conditions other than DRESS syndrome. Six of the 11 patients who had prior systemic corticosteroid exposure were treated for 2-4 days at outpatient clinics or outside hospitals (patients 1, 7, 10, 12, 15, and 17). Upon presentation to UCH and dermatology consultation, corticosteroids were discontinued, and a cyclosporine course was initiated. These patients were switched to cyclosporine due to failure to improve on corticosteroids or to avoid exacerbating concurrent medical comorbidities, such as diabetes or wound infections. In 1 of the 11 cases of prior corticosteroid exposure (patient 3), the patient had been receiving a course of a high dose of prednisone (40 mg on day 1 followed by 80 mg daily) prior to dermatology consultation. Given the increased risk of DRESS syndrome rebound with abrupt discontinuation of the high-dose corticosteroid, the patient was continued on a steroid taper in conjunction with the cyclosporine treatment. In 2 of the 11 cases, patients had a DRESS syndrome recurrence while on a systemic corticosteroid taper (patients 2 and 13). In these instances, the corticosteroid taper was continued in addition to the initiation of cyclosporine due to the risk of DRESS syndrome rebound with abrupt cessation of corticosteroids. In 1 of the 11 cases (patient 6), the patient had received 5 days of systemic corticosteroid for adrenal insufficiency prior to initiation of a cyclosporine course, which was continued along with the corticosteroid course. Finally, in 1 of the 11 cases (patient 18), the patient was on their fifth month of a prednisone taper at the time of DRESS syndrome onset. Given the risk of adrenal insufficiency with abrupt discontinuation of prednisone, the patient was continued on their corticosteroid taper in addition to cyclosporine treatment for the treatment of DRESS syndrome.

In 8 of 19 DRESS syndrome occurrences, cyclosporine initiation occurred without prior corticosteroid exposure. Cyclosporine was the preferred treatment due to concern for prolonged prednisone taper, to avoid extended immunosuppression in the setting of recent or concurrent infection, to restart immunologic treatment of metastatic melanoma, for more rapid improvement of symptoms, and due to previous successful treatment of DRESS syndrome with cyclosporine.

### Outcomes

Of the 19 cases in this case series, 17 patients had a resolution of DRESS syndrome (17/19, 89%) with treatment of cyclosporine. One of the patients who did not have a resolution with cyclosporine was the only patient not hospitalized and was lost to follow-up after initiation of cyclosporine (patient 10). The second patient that did not resolve with cyclosporine (patient 19) was switched to prednisone after 2 days of cyclosporine treatment and had improvement of DRESS syndrome symptoms on prednisone. Of the 17 patients who had a resolution of DRESS syndrome when treated with cyclosporine, 16 (94%) had resolved DRESS syndrome symptoms on discharge when treated with cyclosporine, with an average time to resolution of 2.83 days (Table 1). The patient with symptoms that did not resolve by discharge (patient 4) had clinical resolution prior to their follow-up appointment with an outpatient dermatologist.

**Table 1:** Cyclosporine dosing and outcomes in patients at the University of Colorado Hospital (UCH) diagnosed with drug reaction with eosinophilia and systemic symptoms (DRESS) syndrome.

Patient	Age	Sex	Causative drug	RegiSCAR score^a^	Prior systemic corticosteroid exposure	Cyclosporine dose (orally taken, unless indicated)	Cyclosporine duration (days)	Days to resolution^b^
1	21	M^c^	Carbamazepine	3	Yes – 4 days	2 mg/kg BID^d^	7	1
2	49	M	Carbamazepine	3	Yes – 13 days	2.5 mg/kg BID	5	3
3	48	F^e^	Dabrafenib	2	Yes – 1 day	2 mg/kg Q12 IV	5	1
4	19	F	Phenytoin or oxcarbazepine	2	No	2.5 mg/kg BID	5	9
5	22	F	Phenytoin	3	No	2.5 mg/kg BID	7	1
6	22	M	Phenytoin	5	Yes – 5 days	2.5 mg/kg BID	3	10
7	47	M	Phenytoin	4	Yes – 4 days	2.5 mg/kg BID	5	1
8	75	M	Phenytoin	4	No	1.5 mg/kg BID	3	2
9	75	M	Phenytoin	2	No	2 mg/kg BID	7	1
10	38	F	Empagliflozin/ Metformin HCl tablets (Synjardy)	3	Yes – 3 days	2.5 mg/kg BID	7	N/A^f^
11	43	F	Vancomycin	6	No	0.667 mg/kg Q12 IV	5	3
12	56	F	Vancomycin	5	Yes – 2 days	2.5 mg/kg BID	7	1
13	65	M	Vancomycin	2	Yes – 45 days	2 mg/kg BID	5	3
14	75	M	Vancomycin	3	No	2.5 mg/kg BID	5	2
15	45	M	Vemurafenib	4	Yes – 3 days	2 mg/kg BID	5	2
16	50	M	Vemurafenib	5	No	2 mg/kg BID	5	1
17	51	M	Vemurafenib	3	Yes – 4 days	2 mg/kg BID	7	2
18	53	F	Vemurafenib	3	Yes – 5 months	2 mg/kg IV Q12; 2 mg/kg PO BID	3.5; 1.5 (respectively)	3
19	59	F	Vemurafenib	2	No	2 mg/kg BID	2	5

^a^RegiSCAR: Registry of Severe Cutaneous Adverse Reaction.

^b^“Days to resolution” refers to days after starting cyclosporine to resolution of fever and rash improvement.

^c^M: male.

^d^BID: twice per day.

^e^F: female.

^f^N/A: not applicable.

The median dose of a cyclosporine treatment course was 2 mg/kg twice per day (BID; mean 2.11) with a median treatment duration of 5 (mean 5.26) days. This dose is consistent with the common initial dosing regimen in the treatment of other skin diseases, such as psoriasis. This dosing regimen has been supported by previously published cases, which commonly used 2.5-5 mg/kg daily of oral cyclosporine [2,3,5-9,15,18]. Patient 11 was treated with a decreased dose of cyclosporine (0.667 mg/kg IV every 12 hours) due to treatment with IV as opposed to oral cyclosporine. This dose adjustment was determined with the assistance of pharmacy to account for the differences in bioavailability. Patient 19 had a shortened course of cyclosporine and was switched to prednisone due to adrenal insufficiency. Only 3 of 19 (16%) of DRESS syndrome occurrences resulted in a recurrence after being treated with cyclosporine. Treatment outcomes for patient 10 are unknown due to the loss of follow-up. Average follow-up time for this cohort was 2.3 years. Three patients were lost to follow-up after hospital discharge. Mortality due to DRESS syndrome was 0% (0/19 patients) for patients in this study.

## Discussion

### Principal Results

In total, 17 of 19 (89%) of our cohort were successfully treated with cyclosporine within 5.26 days of treatment initiation on average. Our study supports findings in the literature and increases the number of cases reported by approximately 26%. The only patient that did not have known DRESS syndrome resolution was lost to follow-up. The approach to treating patients with cyclosporine typically started with a 5-day treatment course with progression to a 7-day treatment course if patients had initially responded to cyclosporine but could benefit from additional immunosuppression based on end-organ damage. In contrast, treatment with prednisone typically includes a taper and may last up to 3-6 months in order to prevent recurrence [19]. Therefore, a short course of cyclosporine may be particularly useful in patients with contraindications to long-term immunosuppression or poor medical follow-up.

Furthermore, the low rate of DRESS syndrome relapse following cyclosporine treatment (16%) is promising, as DRESS syndrome frequently involves recurrence, despite discontinuation of the original causative drug. Studies have found up to a 25% recurrence rate in their cohorts treated with the standard of care. The corticosteroid taper has been implicated in some recurrences [20]. In 1 of the 3 cases of recurrence in this study, the second case of DRESS syndrome was thought to result from a different causative agent that occurred 17 months after the first occurrence of DRESS syndrome. The patient had a second recurrence of DRESS syndrome 13 months after their first recurrence, which was due to a retrial of the initial causative agent. The remaining 2 cases of recurrence occurred within the same hospitalization as the initial occurrence of DRESS syndrome. The first of these recurrences involved worsening symptoms 3 days after completion of a cyclosporine course and 1 day after discontinuing methylprednisolone. Notably, the patient had also been receiving methylprednisolone for limbic encephalitis. The second case of these recurrences within the same hospitalization was successfully treated with a longer duration of cyclosporine at a higher dose (2 mg/kg BID for 7 days instead of the initial 1.5 mg/kg for 3 days) without a known subsequent recurrence.

### Limitations

The mean RegiSCAR score of 3.42 was low due to a lack of documentation required to fully calculate a RegiSCAR score. As this was a retrospective study, the RegiSCAR criteria were not used to make the diagnosis, and there are features included in the RegiSCAR criteria that were not evaluated when making the diagnosis. Thus, 12 of 19 (63%) patients had complete data for 6 of the 8 categories [17]. Most patients did not have complete laboratory and imaging data to fully assess organ involvement, particularly for the lung, pancreas, and muscle or heart. Laboratory data for other possible causes of symptoms, including antinuclear antibodies, hepatitis serologies, and evaluation of concomitant mycoplasma or chlamydia pneumonia infection, were not completely evaluated. However, a mean RegiSCAR score of 3.42 is between a “possible case” and “probable case” of DRESS syndrome. Additionally, all patients included in this study were diagnosed with DRESS syndrome by a board-certified dermatologist due to experiencing a suspected drug-related reaction, acute morbilliform rash involving at least 75% body surface area, and internal organ involvement.

This study is limited by its retrospective nature and lack of long-term patient follow-up. Median follow-up time was shorter than expected due to the loss of 3 patients to follow-up and 3 patient deaths due to their primary disease. Additionally, most patients were on a corticosteroid at the time of DRESS syndrome diagnosis. This limitation is likely a consequence of UCH being a tertiary referral center because patients may have already been trialed on a corticosteroid prior to presentation to UCH or dermatology consults and subsequent initiation of cyclosporine. The concomitant use of corticosteroids is a common limitation to previous literature on using cyclosporine in DRESS syndrome [2,3,5,8-11,14-16]. However, the promising results of our 19 cases further support the consideration of cyclosporine in the treatment of DRESS syndrome, particularly when corticosteroids are contraindicated, or the patient may be unable to consistently take a long steroid taper. Further studies that may expand on this data include those with more consistent long-term follow-up with patients, comparison of outcomes between cyclosporine treatment regimens, and studies with larger samples of patients treated with cyclosporine.

### Comparison With Prior Work

Corticosteroids, as opposed to cyclosporine, are the standard of care in the treatment of DRESS syndrome. Therefore, this literature on cyclosporine in the treatment of DRESS syndrome has been limited to primarily small case reports, 1 case-control study with 5 patients treated with cyclosporine compared to 21 patients treated with corticosteroids [4], and 1 retrospective study with 27 patients treated with cyclosporine compared to 53 patients treated with corticosteroids [12]. The previous cases are summarized in the Multimedia Appendix 1 [2-11,13-16]. Data from the retrospective study are not included in the summary, as the data were aggregated and could not be separated into individual cases. The vast majority of these previous studies have suggested that cyclosporine is effective and may lead to rapid clinical resolution of DRESS syndrome [2-12,16]. In the case-control study, the patients treated with cyclosporine were found to have a shorter time to clinical resolution than those treated with corticosteroids [4]. These results are further supported by the retrospective study, in which hospitalization length, treatment length, and time to resolution of DRESS syndrome symptoms were found to be comparable between cyclosporine and corticosteroid treatment groups, while the cyclosporine group had fewer adverse events from treatment [12]. Additionally, in 15 of the 27 cases, cyclosporine was successfully used after symptoms failed to resolve or relapsed with corticosteroid treatment [5-11]. Of the 3 cases in which cyclosporine failed, 2 cases also did not resolve with corticosteroid treatment alone [13,14]. Cyclosporine failed with successful methylprednisolone rescue in only 1 of the 27 cases reported in the literature [15]. There was 1 reported case of recurrence after resolution with the use of cyclosporine. This recurrence was treated with a prolonged taper of both cyclosporine and methylprednisolone [16].

Additionally, the average time to resolution of DRESS syndrome after initiation of cyclosporine was 2.83 days. Our findings are consistent with the case-control study, in which 5 patients treated with cyclosporine had a shorter time to resolution of symptoms compared to patients treated with glucocorticoids (3.5 days versus 5.5 days, respectively) [4]. The discrepancy in time to resolution between treatment with cyclosporine and treatment with prednisone or no treatment, suggests that cyclosporine may have improved clinical resolution compared to the current standard of care.

### Conclusions

The current standard of care for DRESS syndrome includes the removal of the causative agent, supportive therapy, and a course of corticosteroids. However, DRESS syndrome continues to have a mortality rate of around 10% and a recurrence rate of 25% [1,2,20]. Cyclosporine shows promise as an effective treatment of DRESS syndrome with possible reduced rates of relapse, more rapid resolution of symptoms, and shorter treatment courses when compared to the current standard of care. Therefore, cyclosporine may be useful in patients who are unable to tolerate 4-6 weeks of immunosuppression. This study supports the use of a 5-day trial with cyclosporine (2 mg/kg BID) in the treatment of DRESS syndrome as an alternative to corticosteroids, particularly in patients who fail to respond to or are intolerant of corticosteroids. Further research is needed to compare treatment outcomes and long-term complications of cyclosporine to those of corticosteroids. Limitations to the use of cyclosporine as a first-line agent include nephrotoxicity and hepatotoxicity [21,22]. However, in the case of DRESS syndrome, medication courses are shorter than that for corticosteroids, limiting medication exposure compared to the current standard of care.

## Appendix

Multimedia Appendix 1 Summary of the current cases concerning cyclosporine in the treatment of DRESS.

## Abbreviations

BID: twice per day
DRESS: drug reaction with eosinophilia and systemic symptoms
RegiSCAR: registry of severe cutaneous adverse reaction
UCH: University of Colorado Hospital
