# Supplementary material for: Cyclosporine in the Treatment of Drug Reaction With Eosinophilia and Systemic Symptoms Syndrome: Retrospective Cohort Study
Source: JMIR Dermatol. 2023 Jul 20;6:e41391. doi: 10.2196/41391 (PMC10401189; doi:10.2196/41391)
Supplement: Multimedia Appendix 1 [file derma_v6i1e41391_app1.docx]

Table 2. Summary of the current cases concerning cyclosporine in the treatment of DRESS.

| Publication Date | Age | Sex | Causative Drug | Initial Corticosteroid Treatment | Cyclosporine Indication | Cyclosporine Dose | Result |
| --- | --- | --- | --- | --- | --- | --- | --- |
| 6/2003 [5] | 37 | F^a^ | Phenytoin | Prednisolone 40 mg/day; Clobetasol propionate 0.05% | Cushingnoid with persistent disease 1-year post-phenytoin discontinuation | 4 mg/kg/day x 6 months | Resolution |
| 8/2005 [10] | Unk^b^ | Unk^b^ | Vancomycin | Methylprednisolone 100 mg IV four times per day followed by prednisone taper | Persistent DRESS | 100 mg BID x 5 days | Resolution |
| 6/2008 [14] | 29 | F^a^ | Celecoxib | Methylprednisolone 1000 mg IV daily x 5 days followed by prednisone 75 mg daily; subsequent relapse followed by methylprednisolone 1000 mg IV daily x 3 days | Eosinophilic polymyositis | 100 mg daily IV x 1 month concurrent with methylprednisolone 60 mg daily | Eventual recovery following prolonged steroid taper |
| 4/2012 [13] | 56 | F^a^ | Sulfasalazine | Coadministration of high-dose dexamethasone and cyclosporine with prednisone taper | Unk^b^ | Unk^b^ | No resolution, myocarditis within 6 weeks of treatment initiation |
| 11/2016 [7] | 40s | F^a^ | Carbamazepine | N/A^d^ | Unk^b^ | 100 mg BID x 7 days | Resolution |
| 11/2016 [7] | 30s | M^c^ | Minocycline | N/A^d^ | Unk^b^ | 2.5 mg/kg BID x 3 days | Resolution |
| 11/2017 [9] | 48 | M^c^ | Mexiletine | Prednisolone 100 mg daily x 9 days followed by 70 mg daily x 6 days and self-weaning | Relapse within 11 days | 2.5 mg/kg BID x 6 days and subsequent taper x 34 days | Resolution |
| 1/2018 [6] | 25 | F^a^ | Lamotrigine | Patient declined | Declined corticosteroids | 3 mg/kg/day x 7 days | Resolution |
| 1/2018 [6] | 88 | F^a^ | Vancomycin | Patient’s son declined | Declined corticosteroids | 3 mg/kg/day x 7 days | Resolution |
| 10/2018 [2] | 59 | M^c^ | TMP/SMX^e^ | Prednisone 1 mg/kg/day | Worsening DRESS | 5 mg/kg/day x 7 days | Resolution |
| 1/2020 [8] | 66 | M^c^ | TMP/SMX^e^ | Methylprednisolone 1 mg/kg/day IV increased to 1.5 mg/kg/day x 2 days and returned to 1 mg/kg/day | Worsening DRESS despite corticosteroid and IVIG x 2 days | 1.5 mg/kg BID IV concurrent with methylprednisolone 1 mg/kg/day followed by 1 mg/kg twice per day oral with slow taper (concurrent with prednisone taper) x 6 months | Resolution |
| 3/2020 [4] | Unk^b,f^ | Unk^b,f^ | Vancomycin | Unk^b,f^ | Unk^b,f^ | Unk^b,f^ | Initial improvement |
| 3/2020 [4] | Unk^b,f^ | Unk^b,f^ | Vancomycin | Unk^b,f^ | Unk^b,f^ | Unk^b,f^ | Initial improvement |
| 3/2020 [4] | Unk^b,f^ | Unk^b,f^ | Sulfasalazine | Unk^b,f^ | Unk^b,f^ | Unk^b,f^ | Initial improvement |
| 3/2020 [4] | Unk^b,f^ | Unk^b,f^ | Captopril | Unk^b,f^ | Unk^b,f^ | Unk^b,f^ | Initial improvement |
| 3/2020 [4] | Unk^b,f^ | Unk^b,f^ | Diltiazem | Unk^b,f^ | Unk^b,f^ | Unk^b,f^ | Initial improvement |
| 6/2020 [15] | Unk^b^ | F^a^ | Vancomycin | Prednisone 40 mg x 1 dose | Avoid prolonged taper given mild presentation | 1.5 mg/kg BID x 3 days | Worsening symptoms, resolution after switch to corticosteroids |
| 8/2021 [3] | 45 | M^c^ | Cotrimoxazole | average 0.77 mg/kg/day x 27 days | Relapse after taper | 2.47 mg/kg/day x 90 days with gradual steroid taper x 6 weeks | Resolution |
| 8/2021 [3] | 15 | M^c^ | Amoxicillin | average 0.4 mg/kg/day x 30 days | Relapse after taper | 0.53 mg/kg/day x 84 days | Resolution |
| 8/2021 [3] | 39 | F^a^ | Etodolac | average 0.83 mg/kg/day x 24 days | Relapse after taper | 1.92 mg/kg/day x 111 days | Resolution |
| 8/2021 [3] | 40 | F^a^ | Amoxicillin | average 0.5 mg/kg/day x 29 days | Persistent DRESS | 1.66 mg/kg/day x 18 days | Resolution |
| 8/2021 [3] | 75 | M^c^ | Zonisamide | average 0.44 mg/kg/day x 47 days | Relapse and adrenal insufficiency with taper | 2.21 mg/kg/day x 75 days | Resolution |
| 8/2021 [3] | 35 | M^c^ | Dapsone | average 0.71 mg/kg/day x 21 days | Relapse with taper | 2.63 mg/kg/day x 90 days | Resolution |
| 8/2021 [3] | 43 | F^a^ | Sulfasalazine | Average 0.37 mg/kg/day x 23 days | Relapse with taper | 1.49 mg/kg/day x 114 days | Resolution |
| 8/2021 [3] | 21 | F^a^ | Amoxicillin | Average 0.61 mg/kg/day x 22 days | Persistent DRESS | 1.66 mg/kg/day x 27 days | Resolution |
| 2/2022 [16] | 15 | F^a^ | Valproic Acid | Methylprednisolone 1 mg/kg/day divided every 12 hours | Persistent DRESS | 100 mg twice per day x 3 days | Resolution with recurrence |
| 3/2023 [11] | 30s | F^a^ | Sulfasalazine | Prednisolone 50 mg daily, tapered by 5mg every 4 weeks | Relapse with taper | 5 mg/kg/day | Resolution |

^a^ F, Female

^b^ Unk, Unknown due to not specified in publication

^c^ M, Male

^d^ N/A, Not applicable

^e^ TMP/SMX, Trimethoprim-Sulfamethoxazole

^f^ Publication from 3/2020 did not differentiate between the 5 cases. Mean age of cohort was 43 with 80% females. 3 patients had contraindication to corticosteroid use. Mean length of treatment with cyclosporine was 12.5 days. 2 patients discontinued cyclosporine due to adverse effects.
